# Direct targeting of host microtubule and actin cytoskeletons by a chlamydial pathogenic effector protein

**DOI:** 10.1242/jcs.263450

**Published:** 2024-09-06

**Authors:** Mona Höhler, Abel R. Alcázar-Román, Katharina Schenk, Mac Pholo Aguirre-Huamani, Corinna Braun, Rafat Zrieq, Katja Mölleken, Johannes H. Hegemann, Ursula Fleig

**Affiliations:** ^1^ Eukaryotic Microbiology, Heinrich-Heine-University, 40225 Düsseldorf, Germany; ^2^ Institute of Functional Microbial Genomics, Heinrich-Heine-University, 40225 Düsseldorf, Germany; ^3^ Department of Public Health, College of Public Health and Health Informatics, University of Haʹil, Ha′il City 2440, Saudi Arabia; ^4^ Applied Science Research Centre, Applied Science Private University, Amman 11931, Jordan

**Keywords:** Cytoskeleton, Microtubules, Pathogenic bacteria, Chlamydia

## Abstract

To propagate within a eukaryotic cell, pathogenic bacteria hijack and remodulate host cell functions. The Gram-negative obligate intracellular Chlamydiaceae, which pose a serious threat to human and animal health, attach to host cells and inject effector proteins that reprogram host cell machineries. Members of the conserved chlamydial TarP family have been characterized as major early effectors that bind to and remodel the host actin cytoskeleton. We now describe a new function for the *Chlamydia pneumoniae* TarP member CPn0572, namely the ability to bind and alter the microtubule cytoskeleton. Thus, CPn0572 is unique in being the only prokaryotic protein that directly modulates both dynamic cytoskeletons of a eukaryotic cell. Ectopically expressed GFP–CPn0572 associates in a dose-independent manner with either cytoskeleton singly or simultaneously. *In vitro*, CPn0572 binds directly to microtubules. Expression of a microtubule-only CPn0572 variant resulted in the formation of an aberrantly thick, stabilized microtubule network. Intriguingly, during infection, secreted CPn0572 also colocalized with altered microtubules, suggesting that this protein also affects microtubule dynamics during infection. Our analysis points to a crosstalk between actin and microtubule cytoskeletons via chlamydial CPn0572.

## Teaser

**Summary:** Identification of a protein from the intracellular pathogenic *Chlamydia* that targets both the actin and microtubule cytoskeletons.

## INTRODUCTION

*Chlamydia pneumoniae* (*Cpn*) is an important obligate intracellular bacterial pathogen, which infects lung epithelial cells, causing human respiratory diseases. It is responsible for 10% of community-acquired pneumoniae and 5% of bronchitis, pharyngitis and sinusitis cases (Grayston et al., 1995). Moreover, *Cpn* is associated with chronic obstructive pulmonary disease, asthma, atherosclerotic cardiovascular diseases and lung cancer (Campbell and Hahn, 2020). All chlamydial and chlamydia-related species undergo a very specific, biphasic infection cycle initiated by the infectious, extracellular and non-dividing elementary bodies (EBs), which invade host cells, and the intracellular metabolically active reticulate bodies (RBs), which replicate inside membrane vacuoles termed inclusions (Stelzner et al., 2023; Bayramova et al., 2018).

For obligate intracellular pathogens, host cell entry is of utmost importance. To ensure internalization Chlamydiae use a complex repertoire of bacterial adhesin – host cell receptor interactions as well as effector proteins translocated by the type-III secretion system (T3SS) (Nans et al., 2015). *Cpn* adhesins on the EB cell surface, such as OmcB, Pmp proteins and LipP, interact with host cell plasma membrane surface structures and these interactions are essential for chlamydial internalization (Wuppermann et al., 2008; Mölleken et al., 2013; Galle et al., 2019; Becker and Hegemann, 2014). Equally important for EB entry are the *Cpn* T3SS-mediated effector proteins such as SemC and SemD, which upon secretion into the host cell cytosol, bind and reshape the host cell plasma membrane underneath the adhered EB and recruit endocytotic proteins to support EB uptake (Hänsch et al., 2020; Spona et al., 2023).

A major target for soluble secreted effector proteins, such as the conserved TarP family, is the host cell cytoskeleton, which is composed of F-actin, microtubules (MTs), intermediate filaments and septins (Hohmann and Dehghani, 2019; Goodson and Jonasson, 2018; Svitkina, 2018; Herrmann and Aebi, 2016; Woods and Gladfelter, 2021). MTs are the most dynamic feature of the cytoskeleton of the cell, consisting of highly dynamic polar filaments that switch between phases of polymerization and depolymerization. These alterations of MT dynamics are controlled by the availability of α/β-tubulin dimers and by the association of a vast array of MT-associated proteins (MAPs) that modulate MT dynamics, especially from the MT plus-end (Meier et al., 2024). Additionally, the composition of a MT filament in terms of its different tubulin isotypes alters its dynamic parameter as does the presence of an array of post-translational modifications (McKenna et al., 2023).

Thus, the MTs even within one cell can have very different orientations and dynamics from each other (Stone et al., 2008; Tanenbaum et al., 2014; Feng et al., 2019), which can be altered quickly in time and space depending on need.

All cytoskeletal structures form a complex interacting meshwork involved in processes such as endocytosis, cell division, intracellular transport, motility, force transmission, reaction to external forces, adhesion and preservation, and adaptation of cell shape. The actin–MT crosstalk is particularly important for many of these processes (Dogterom and Koenderink, 2019). Importantly, the continued extensive crosstalk between these two cytoskeletons for basic biological functions has come into focus. An array of common regulators has been identified that co-modulate these cytoskeletons in time and space, pointing to an integrated system instead of the two separate actin and MT components of the cytoskeleton (Dogterom and Koenderink, 2019). This fact is not usually taken into consideration when the modulation of the actin cytoskeleton by bacterial pathogens is studied. In fact, typical protocols used for synchronous early chlamydia infection utilize spinning and 4°C incubation, which destroy the MT cytoskeleton. Thus, although the modulation of the host actin cytoskeleton by bacterial pathogens is well studied, the connection to the MT cytoskeleton is missing as is a thorough analysis of the MT impact on bacterial infection (Stevens et al., 2006; Colonne et al., 2016; Haglund and Welch, 2011).

All four cytoskeletal structures have been found to be associated with a *C. trachomatis* (*Ctr*) infection (Clifton et al., 2004; Volceanov et al., 2014; Al-Zeer et al., 2014; Kumar and Valdivia, 2008), but an understanding of their molecular function and role during infection is fragmentary, although the properties of specific chlamydial proteins in modulating MT dynamics have been analyzed extensively (Campanacci et al., 2019). It is known that early during infection, *Ctr* traffics along MTs to the MT-organizing center (MTOC; which is close to the nucleus) of the host cell to establish its intracellular niche, the inclusion (Grieshaber et al., 2003; Mital et al., 2015). Here the inclusion initiates and maintains a close association with the MTOC (also known as the centrosome) (Luis et al., 2023). A *Ctr* infection leads to MT dependent mitotic alterations (Grieshaber et al., 2006; Knowlton et al., 2011). Recently, it was shown that the *Ctr*-specific T3-secreted soluble effector protein CteG interacts with a key structural component of centrosomes and the absence of CteG impairs chlamydia's ability to replicate efficiently in primary cervical cells and in a murine model of *Ctr* infection (Steiert et al., 2023).

The important role of the MT cytoskeleton during *Cpn* infection is just beginning to emerge. Recently, we carried out a functional screen of 116 *Cpn* proteins and identified 13 *Cpn* effector proteins that modulate MT dynamics in different manners (Wevers et al., 2023). Six of the 13 proteins have a putative homolog in *Ctr*, seven have homologs in other Chlamydia and five proteins are unique to *Cpn*. Interestingly, one of the identified *Cpn* modulators altering the MT cytoskeleton is CPn0065, which has a *Ctr* homolog named IncM. A recent publication uncovers the role of IncM in MT modulation, suggesting that the hijacking of the MT cytoskeleton during a chlamydial infection might be a common trait (Luis et al., 2023).

The earliest known chlamydial effector, *Ctr* TarP is T3SS-translocated into the host cell cytosol within the first 5 min post infection, where it is directly involved in actin polymerization. Its proline-rich domain allows TarP oligomerization, which results in high G-actin concentrations mediated by the TarP actin-binding domain (ABD), facilitating direct F-actin polymerization (Jewett et al., 2010). This is indirectly supported by TarP binding to the actin adaptor protein vinculin, which also promotes F-actin recruitment (Thwaites et al., 2015). TarP homologs can be found in all species within the family Chlamydiaceae (Lutter et al., 2010). The *Cpn* TarP family member CPn0572 and *Ctr* TarP share the same actin nucleation activity *in vitro* and show a similar actin-recruiting activity early in infection *in vivo* (Jewett et al., 2010; Zrieq et al., 2017). In fact, both *Ctr* TarP and CPn0572 bind F-actin *in vitro* and *in vivo* (Jiwani et al., 2013; Zrieq et al., 2017). In addition, from previous publications on CPn0572, it is known that CPn0572 is present in EBs as mRNA (Mäurer et al., 2007) and as protein in EB lysates (Jewett et al., 2010; Zrieq et al., 2017), and is detected via antibody 15 min after infection colocalizing with actin and EBs (Zrieq et al., 2017). Moreover, CPn0572 also exhibits actin-related activities unknown for TarP. The CPn0572 C-terminal part stabilizes host F-actin by displacing members of the F-actin-severing factor cofilin family (Zrieq et al., 2017) and the N-terminal part of CPn0572 appears to contain a domain that inhibits the ability of the ABD to colocalize with actin (Braun et al., 2019).

These differences probably reflect the dissimilar requirements for the infection of the diverging anatomical sites: pulmonary epithelia versus eye and genital tract epithelia.

However, the most surprising difference between both proteins became apparent in their localization profile upon ectopic expression in human epithelial cells – a TarP–GFP fusion protein exclusively localized to distinct actin-containing aggregates, whereas a GFP–CPn0572 fusion protein colocalized with smaller actin aggregates and with filamentous structures emanating from them showing a continuous colocalization with distinct actin fibers. Remarkably, CPn0572 also localized to filamentous structures that were devoid of actin (Zrieq et al., 2017). The nature of these CPn0572-positive fibers and their relationship to other cellular structures remained obscure (Zrieq et al., 2017; Braun et al., 2019).

In this paper, we identified a new and unique function for *Cpn* TarP members. We found that CPn0572 can directly bind to MTs *in vitro* and *in vivo* and modulate MTs upon ectopic expression and during infection. The finding that CPn0572 can bind to either the MT and/or the actin cytoskeleton, and that the loss of the MT cytoskeleton alters its actin modulating properties suggests that CPn0572 might be able to alter the MT–actin crosstalk during early infection.

## RESULTS

### The actin modulator CPn0572 also colocalizes with the MT cytoskeleton

**Fig. 1.**
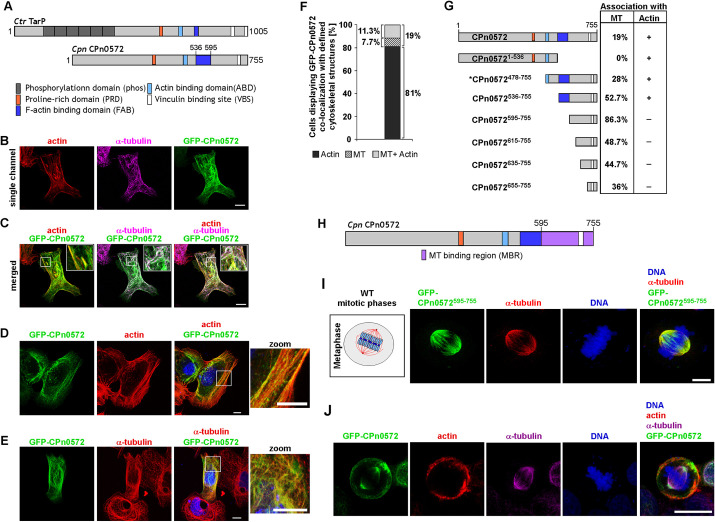
**CPn0572 associates with the interphase and mitotic MT cytoskeleton.** (A) Schematic representation of the *Ctr* TarP protein and full-length *Cpn* CPn0572 protein. Previously identified domains are shown: phosphorylation domain (phos), dark gray boxes; proline-rich domain (PRD), orange box; G-actin binding domain (ABD), light blue box; F-actin-binding domain (FAB), dark blue box; and vinculin-binding site (VBS), white box (Zrieq et al., 2017; Braun et al., 2019). (B) Representative confocal images of HEp-2 cells expressing GFP-CPn0572 (green) for 24 h prior to fixation. Actin was visualized with Rhodamine–phalloidin staining (red) and MTs with anti-α-tubulin antibody. As secondary antibody anti-mouse-IgG conjugated to Alexa Fluor 647 was used (magenta). (C) Merged images of indicated single channels shown in (B). White boxes show enlargements. (D) Representative confocal images of HEp-2 cells transfected with plasmid expressing GFP–CPn0572 (green) for 24 h, prior to fixation. Shown is an exclusive predominant association of GFP–CPn0572 with actin. Actin was visualized with Rhodamine-phalloidin staining (red). (E) Shown is a predominant association of GFP–CPn0572 with interphase MTs visualized with anti-α-tubulin antibody (red). DAPI was used to visualize DNA (blue). (F) Quantification of HEp-2 cells transfected with a GFP–CPn0572 plasmid 24 h prior to fixation. Exclusive colocalization of GFP–CPn0572 with actin was scored in 81% (black) cells, whereas in 11% cells analyzed GFP–CPn0572 localized to both MTs and actin structures (dotted). In 7.7% cells, an exclusive GFP–CPn0572 localization to MT structures was observed (striped). Data represent a mean of three experiments, *n*=100 cells/experiment. (G) Diagrammatic representation of CPn0572 variants. Quantification of cells expressing GFP–CPn0572 variants and association with the MT cytoskeleton as a percentage (%) of cells counted. Association with the actin cytoskeleton is depicted as+(actin association) or−(no actin association). When no actin association was observed (four bottom CPn0572 variants), CPn0572 was either associated with MT structures (percentage given) or showed a non-specific cytoplasmic staining. * marks deletion variant with MT and actin phenotypes differing from the other protein variants (visualized in Fig. S1, dot-like and curved-fiber phenotypes concentrated in proximity to the nucleus). (H) Schematic representation of full-length CPn0572 including the newly defined MT-binding region from aa 595–755 (purple box). (I) Confocal images of U2OS cells transfected with GFP–CPn0572^595-755^ plasmid for 18 h. Mitotic metaphase is shown diagrammatically on the left. MTs were visualized by anti-α-tubulin antibody staining (red) and DNA with DAPI (blue). (J) Representative confocal image of mitotic U2OS cell expressing full length GFP-CPn0572 (green) full length for 18 h. MTs were visualized by using anti-α-tubulin antibody (magenta), actin with rhodamine-phalloidin staining (red) and DNA with DAPI. In A and G, the numbers indicate amino acid positions. For B–E, I and J images shown are representative of three or more repeats. Scale bars: 10 µm.

Previously, we have shown that ectopically expressed GFP–CPn0572 colocalized predominantly with the actin cytoskeleton of HEK293T cells as has been demonstrated for other members of the chlamydial TarP family (Braun et al., 2019; Zrieq et al., 2017; Clifton et al., 2004) (Fig. 1A). However, we found that in some cells CPn0572 also localized to non-actin microfilaments (Zrieq et al., 2017). To determine whether the non-actin localization might in fact be association with the MT cytoskeleton, HEp-2 cells were transfected with a plasmid expressing full-length GFP–CPn0572 (Fig. 1A) and fixed cells were stained with Rhodamine–phalloidin (actin) and an anti-α-tubulin antibody. Indeed, in 11% of cells, the chlamydial protein colocalized with both the MT and actin cytoskeleton (Fig. 1B and merged images in Fig. 1C; quantification in Fig. 1F). GFP–CPn0572 exclusive colocalization with the actin cytoskeleton (Fig. 1D) was observed in 81% cells, whereas exclusive colocalization with the MT cytoskeleton was scored for ∼8% of HEp-2 cells. (Fig. 1E; quantification in Fig. 1F). We conclude that CPn0572 can associate with both dynamic cytoskeletons of an interphase mammalian cell, although it is unclear what determines the preferred association with both or an individual cytoskeletal structure.

To identify the CPn0572 MT-binding region, we generated a number of GFP-tagged CPn0572 truncated variants and assayed their ability to associate with the actin and/or MT cytoskeleton in HEp-2 cells. The N-terminal variant CPn0572^1–536^ showed exclusive actin colocalization, indicating that the MT-binding region must be present in the C-terminal part of the protein (Fig. 1G). C-terminal variants of this region showed that the absence of the G-actin- and F-actin-binding domains increased the number of cells where the variant protein showed exclusive colocalization with MTs (Fig. 1A,G). In particular, in 86% of cells, the variant GFP–CPn0572^595-755^ localized with the MT cytoskeleton. The remaining transfected cells showed a non-specific cytoplasmic staining but no actin staining. Other C-terminal CPn0572 variants also showed no actin colocalization but a reduced MT colocalization frequency in comparison to CPn0572^595-755^ although they were expressed in higher amounts (Fig. 1G; Fig. S1A,B). Thus, the MT-binding region (MBR) of CPn0572 is confined to the C-terminal 595–755 amino acids (Fig. 1H).

To determine whether the chlamydial protein associated exclusively with interphase MTs or was also able to colocalize with other MT structures, such as a mitotic spindle, human osteosarcoma U2OS cells were transfected with a plasmid-encoding GFP–CPn0572^595-755^ for 18 h. U2OS cells are frequently used for mitotic analysis and the chosen CPn0572 variant was used as it showed a predominant association with interphase MTs. Indeed GFP–CPn0572^595-755^ colocalized with spindles during all stages of mitosis (Fig. 1I; Fig. S2). To determine whether full length GFP–CPn0572 was also able to associate with the spindle, we expressed this protein in U2OS mitotic cells and also found that the protein associated with spindle MTs but was also able to localize to the cortical actin network adjacent to the plasma membrane (Fig. 1J).

### Association with the MT cytoskeleton is restricted to *Cpn* TarP members

**Fig. 2.**
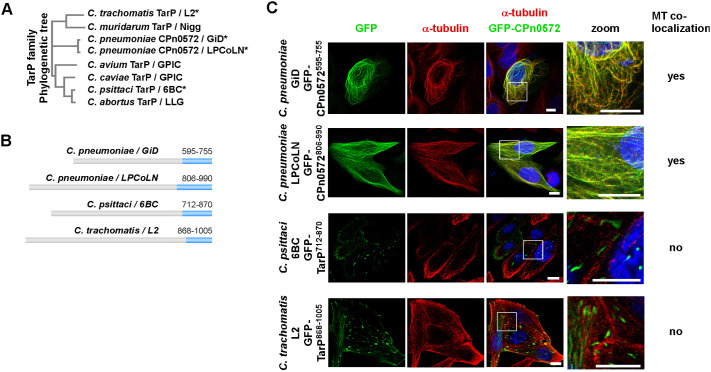
***Cpn* TarP proteins from human and animal isolates can associate with the MT cytoskeleton.** (A) Phylogenetic tree of members of the TarP protein family from different chlamydial species with the corresponding isolate indicated. Asterisks mark the TarP members selected for transfection experiment show in C. Phylogenetics were undertaken with Clustal Omega. (B) Schematic illustration of TarP family members and the C-terminus (blue) used for generation of GFP-fusion variants. (C) Representative confocal fluorescence images of HEp-2 cells expressing GFP-tagged versions of C-terminal fragments of the indicated TarP family members. Determination of colocalization with MTs is shown on the right. Cells were transfected with indicated plasmids for 18 h. MTs were visualized using anti-α-tubulin antibody (red) and DNA with DAPI (blue). White boxes show enlargements. Images shown are representative of three repeats. Scale bars: 10 µm.

We next determined whether association with the MT cytoskeleton is a conserved feature of other CPn0572/TarP family members. A comparison of 23 CPn0572 amino acid sequences from different *Cpn* isolates (Table S1) revealed that 20 out of 23 were identical to the CPn0572 sequence found in the GiD strain used in this study (Fig. S3A). The animal-derived *Cpn* isolate DC9 had a number of amino acid alterations, whereas the animal-derived isolates B21 and LPCoLN showed extensive differences. These include single amino acid (aa) changes, a large insertion of 211 amino acids, and a 24-amino-acid insertion in the region from aa 595 to aa 755, which we identified as the CPn0572 MBR from *Cpn* GiD (Fig. 2B; Fig. S3A). Thus, it was interesting to determine whether the insertion affected MT binding of this region. Furthermore, we selected *Ctr* (L2) TarP and *C. psittaci* (6BC) TarP family members for analysis, as these belong to different clades to *Cpn* (Fig. 2A). The selected CPn0572/TarP members were aligned to identify amino acid regions with homology to CPn0572^595-755^ from strain GiD. A region of high similarity identified in GiD, LPCoLN and 6BC (red letters in Fig. S3B) was utilized to design N-terminal GFP fusions of these C-terminal domains (Fig. 2B, marked in blue). In the case of *Ctr* L2, no clear homology was found. Nevertheless, we tested a GFP–TarP^868-1005^ fusion protein for its ability to bind MTs (Fig. 2B). Expression of these four variants in HEp-2 cells and subsequent microscopy analysis showed that the *Cpn* LPCoLN variant was also able to colocalize with MTs (Fig. 2C), whereas the C-terminal constructs derived from *Ctr* and *C. psittaci* family members did not (Fig. 2C). The latter two constructs instead colocalized with vinculin (Fig. S3C). Thus, the ability of TarP members to associate with the host MT cytoskeleton is a feature conserved in *Cpn* members.

### CPn0572 expression in epithelial cells leads to an altered MT cytoskeleton

**Fig. 3.**
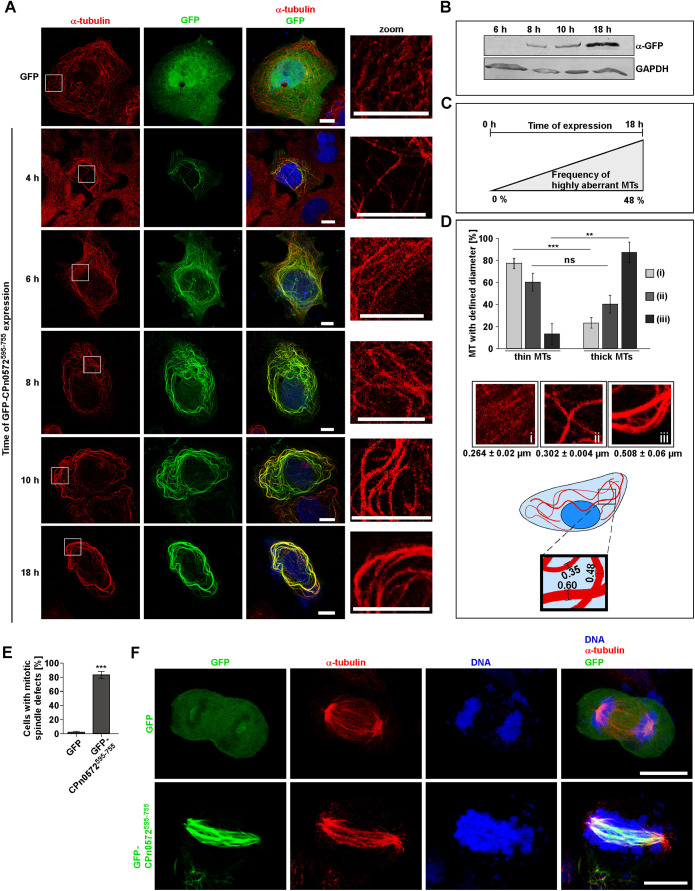
**Severity of alteration of MT structure correlates with time of CPn0572^595-755^ expression.** (A) Representative confocal fluorescence images of HEp-2 cells transfected with a plasmid encoding GFP for 18 h (top panels) or GFP–CPn0572^595-755^ for the indicated times prior to fixation. MTs were visualized with anti-α-tubulin antibody (red) and DNA with DAPI (blue). Scale bars: 10 µm. (B) Western blot analysis of HEp-2 cells expressing GFP-CPn0572^595-755^ for the indicated time points. Protein extracts were separated on a 10% SDS-PAGE followed by Western blot analysis. Western blot was probed with anti-GFP or anti-GAPDH antibodies. Blot shown is representative of two repeats. (C) Explanatory cartoon to show co-dependency of GFP–CPn0572^595-755^ expression levels and the increase of aberrant MTs (only the 0 h and 18 h time points represent actual measurements). (D) Quantification (mean±s.e.m.) of MT phenotypes shown in A. Typical examples of MTs phenotypes are shown for (i) GFP-expressing control cells (18 h), (ii) GFP–CPn0572^595-755^-expressing cells (6 h) and (iii) GFP–CPn0572^595-755^-expressing cells (18 h). MT thickness was determined by calculating the average diameter measured at three different positions of a single MT bundle using ImageJ. Thin MTs were defined as MTs with <0.302 µm in diameter; thick MTs were defined as MTs with ≥0.302 µm in diameter. *n*=3 cells (30 MTs measured per condition). ***P<0.001; **P<0.005; ns, not significant (two-tailed unpaired Student's *t*-test). (E) Quantification (mean±s.e.m.) of the percentage of GFP- or GFP–CPn0572^595-755^-expressing mitotic U2OS cells with spindle defects. *n*=3 independent experiments each representing 50 cells. ***P<0.001 (two-tailed unpaired Student's *t*-test). (F) Confocal images of U2OS cells transfected with a plasmid encoding GFP or GFP-CPn0572^595-755^ for 18 h. Top images show a GFP-expressing mitotic cell in anaphase. Bottom images show a representative example of the main spindle defect observed in GFP–CPn0572^595-755^*-*expressing cells. MTs were visualized by using an α-tubulin antibody (red) and DNA was stained with DAPI (blue). Images shown are representative of three repeats. Scale bars: 10 µm.

In the above analysis, we noticed that the MT cytoskeleton in CPn0572-expressing cells was often aberrant, for example, showing abnormally thick interphase MT bundle structures, which were not observed in control cells expressing only GFP. To better characterize this phenotype, we analyzed interphase MTs in HEp-2 cells transfected with GFP–CPn0572^595-755^ for various times prior to fixation (Fig. 3A). Interestingly, MT staining revealed a correlation between the amount of GFP–CPn0572^595-755^ present and the number of abnormal, thicker MTs (Fig. 3A–C). The diameter of the MT bundles increased from ∼0.26 µm (control) to ∼0.51 µm 18 h after transfection (Fig. 3D). Thus, expression of GFP–CPn0572^595-755^ results in abnormally thick interphase MTs, as has been observed upon overexpression of a MAP (Fong et al., 2013).

Next, we assayed whether the mitotic spindle was also altered upon expression of CPn0572^595-755^ by transfecting U2OS cells with a plasmid encoding this CPn0572 variant for 18 h. We found that, in contrast to U2OS control cells expressing only GFP, 83% of the spindles were abnormal (Fig. 3F, quantified in Fig. 3E). A number of abnormal spindle phenotypes was scored, the most prominent, had nearly 40% of mitotic cells with elongated, irregular anaphase spindle structures with misaligned chromosomes, indicating a defect in bipolar chromosome–spindle attachment (Fig. 3F).

Thus, CPn0572^595-755^ associates with interphase and mitotic MT structures, which leads to aberrant MTs with an altered function.

### Ectopic expression of CPn0572 variants in human cells implies that the MT function of the protein plays a role in chlamydial early infection

TarP family members have been classified as important chlamydial effector proteins that modulate the host actin cytoskeleton. With our discovery that *Cpn* members also colocalize with and modulate the MT cytoskeleton, it was of interest to determine whether this property affected chlamydial infection. Furthermore, we wanted to analyze the importance of the actin versus the MT-modulating effect by assessing the impact on infections of full-length CPn0572, which mainly associates with cellular actin structures but can also colocalize with MTs, and CPn0572^595-755^, which associates with MTs only. We also expressed the N-terminal variant CPn0572^1-536^ that only binds actin.

**Fig. 4.**
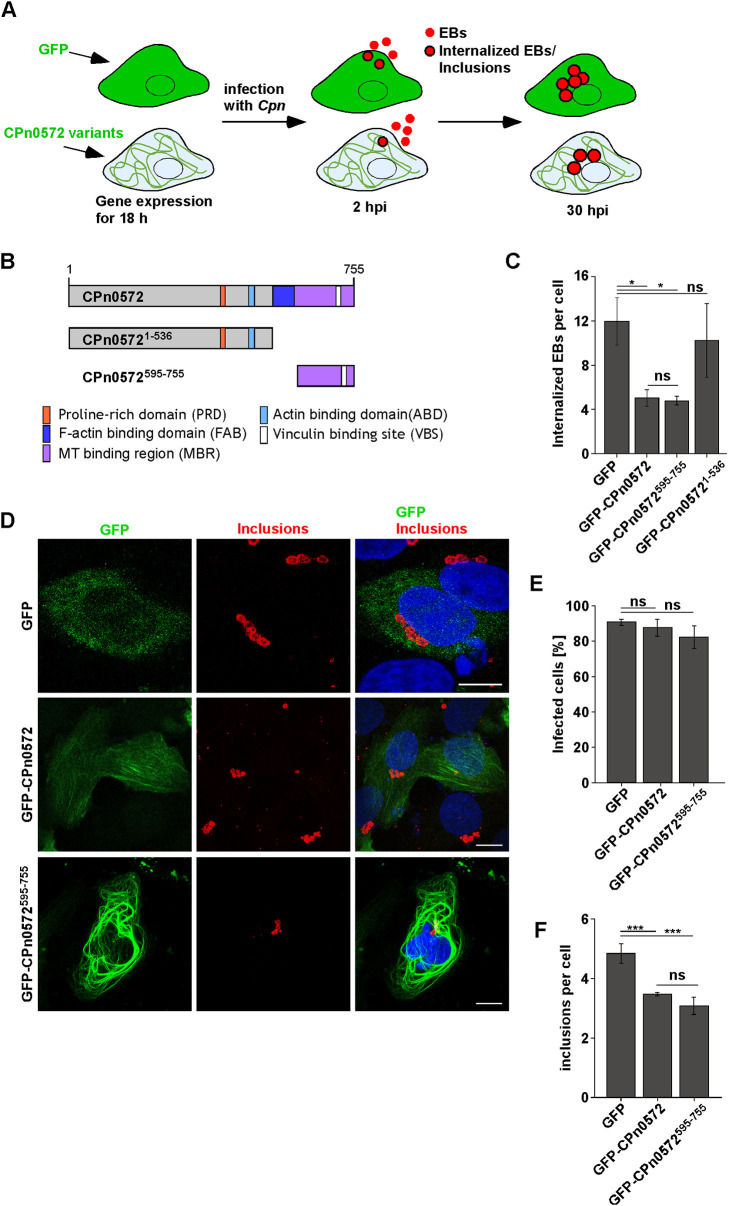
**CPn0572-induced MT modulation attenuates *Cpn* infection.** (A) Schematic representation of the experimental setup. (B) Schematic representation of the used CPn0572 protein variants. (C) Quantification of the number of internalized *Cpn* EBs 2 h post infection (MOI=15). Prior to infection, cells were transfected with the GFP or GFP-tagged CPn0572 variants for 18 h. After paraformaldehyde fixation, cells were not permeabilized to stain EBs that were not internalized. *n*=3, each representing up to 130 EBs. *P<0.05; ns, not significant (two-tailed unpaired Student's *t*-test). (D) Visualization of *Cpn* inclusions by confocal fluorescence analysis of HEp-2 cells transfected with plasmids encoding GFP or GFP-tagged CPn0572 variants for 18 h, followed by infection with *Cpn* EBs (MOI=5) for 30 h. Inclusions were visualized with the *Cpn* inclusion membrane protein antibody anti-CPn0147 (red) and DNA with DAPI (blue). Scale bars: 10 µm. (E) Quantification of the number of infected HEp-2 cells (≥1 inclusion) transfected and infected as described in D. (F) Quantification of *Cpn* inclusions per cell in HEp-2 populations transfected with indicated plasmids for 18 h prior to *Cpn* infection (MOI=5). For E and F; *n*=4–6 samples each representing 100 GFP-positive cells. ***P<0.001; ns, not significant (two-tailed unpaired Student's *t*-test). All error bars denote ±s.e.m.

GFP-tagged full-length CPn0572, the N-terminal variant CPn0572^1-536^ and the C-terminal variant CPn0572^595-755^ were expressed in HEp-2 cells for 18 h prior to *Cpn* infection and the number of internalized EBs was scored after 2 h and the number of inclusions was scored after 30 h. Fig. 4A is a diagrammatic representation of the experimental set-up; Fig. 4B shows the CPn0572 variants used. Although expression of CPn0572 or CPn0572^595-755^ had no visible effect on HEp-2 cells, the expression of CPn0572^1-536^ in HEp-2 led to cells with typical apoptotic features after 48 h, demonstrating that CPn0572^1-536^ expression was toxic. Thus, for this variant, we could only determine the number of internalized EBs 2 h post infection.

The number of internalized EBs in either CPn0572 and CPn0572^595-755^ expressing cells was similar but significantly lower than those in control cells or CPn0572^1-536^ expressing cells (Fig. 4C). Thus, the modulation of the MT cytoskeleton by ectopic expression of this protein affects invasion efficiency of the pathogen. In addition, the number of infected GFP-, GFP–CPn0572- or GFP–CPn0572^595-755^-expressing HEp-2 cells was comparable (Fig. 4E), but the number of chlamydial inclusions per cell was reduced in the presence of GFP–CPn0572 or GFP–CPn0572^595-755^ (Fig. 4D,F).

### CPn0572 expressed in and secreted by *Ctr* modulates MT structures during infection

**Fig. 5.**
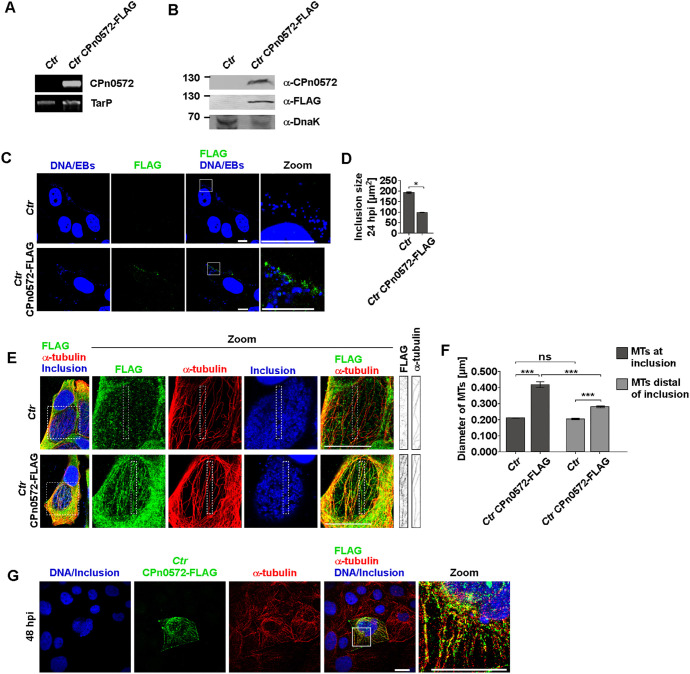
**CPn0572 associated with MTs during chlamydial infection impacts infection efficiency.** (A) PCR and (B) western blot verification of *Ctr* expressing CPn0572–FLAG. For B cells infected with *Ctr* or *Ctr* transformed with plasmid encoding CPn0572–FLAG (pMH16) for 48 h were lysed and proteins were separated on 10% SDS-PAGE. For following western blot analysis anti-CPn0572 (generated in our lab), anti-FLAG and anti-DnaK antibodies were used. Images shown are representative of one (A) or two (B) repeats. (C) Confocal images of HEp-2 cells infected with *Ctr* or *Ctr* transformed with plasmid encoding CPn0572–FLAG for 15 min. CPn0572–FLAG was visualized with anti-FLAG antibody (green) and DNA with DAPI (blue). (D) Quantification (mean±s.e.m.) of inclusion size [µm^2^] 24 hpi of *Ctr* or *Ctr* transformed with plasmid encoding CPn0572–FLAG. *n*=2, each representing 30 cells. *P<0.05 (two-tailed unpaired Student's *t*-test). (E) Representative images from three independent experiments showing single focal planes of HEp-2 cells infected with *Ctr* or *Ctr* transformed with plasmid encoding CPn0572–FLAG (MOI=0.5, 48 hpi). Cells were treated with 0.5% Triton X-100 for 30 s and fixed with 0.5% glutaraldehyde. Images show a focal plane between the inclusion and the plasma membrane. (F) Quantification (mean±s.e.m.) of the diameter of MTs measured at the inclusion or distal of the inclusion in cells infected with *Ctr*- or CPn0572-expressing *Ctr*. MT diameter was measured by measuring the diameter at three different positions of one MT fiber using ImageJ. Data represent measurements of MT diameter for 10 MTs (*n*=4 cells). ***P<0.001; ns, not significant (two-tailed unpaired Student's *t*-test). (G) Representative images from three repeats of Caco-2 cells infected with *Ctr* transformed with plasmid encoding CPn0572–FLAG for 48 h. For E and G, CPn0572–FLAG was visualized with anti-FLAG (green), MTs with anti-α-tubulin antibody (red) and eukaryotic and chlamydial DNA with DAPI (blue). For C, E and G, white boxes show enlargements (zoom). Scale bars: 10 µm.

As ectopic expression of CPn0572 in epithelial cells affects MT structures and *Cpn* internalization, we next tested whether chlamydial expression of CPn0572 during infection would also modulate MT structures. As generation of a CPn0572 antibody useable for advanced immunofluorescence analysis was unsuccessful, we chose plasmid-borne expression of a tagged CPn0572 version in *Ctr* for the analysis. In contrast to *Cpn*, the plasmid expression system in *Ctr* is well established. A plasmid carrying full-length CPn0572 with a C-terminal FLAG tag under control of the constitutive IncD promoter (Agaisse and Derré, 2013; Belland et al., 2003) was transformed in *Ctr* L2 (Fig. 5A). In general, the developmental cycle was slower when CPn0572 was present. Thus, HEp-2 cells infected with CPn0572–FLAG-expressing *Ctr* transformants expressed CPn0572–FLAG for 48 h post infection (hpi) (Fig. 5B). To determine whether the protein is translocated into the host-cell cytoplasm early in infection, infected HEp-2 cells were fixed at 15 min post infection and analyzed by immunofluorescence microscopy. DNA-stained *Ctr* EBs were found to lie close to, or to partially overlap with, CPn0572–FLAG signals indicative of CPn0572 secretion (Fig. 5C). At 24 hpi, development of the CPn0572-expressing inclusion was affected, with the inclusion size reduced by more than 50% compared to untransformed control inclusions (Fig. 5D). Analysis of the subcellular localization of the CPn0572–FLAG fusion protein by immunofluorescence confocal microscopy at 48 hpi revealed a dense cytosolic localization exhibiting numerous filamentous structures, whereas the untransformed control cells only showed background cytosolic signals (Fig. 5E). Interestingly, a detailed look at focal planes lying between the inclusion membrane and the plasma membrane identified filamentous-like CPn0572 structures that were not associated with actin (Fig. S4) but rather with MT structures (Fig. 5E; Fig. S4), a phenotype not seen in *Ctr* control cells (Fig. 5E, see zoom and details of the areas denoted by the white boxes for FLAG and α-tubulin signals). Observing focal planes located between the inclusion membrane and the plasma membrane allowed us to detect clear individual MTs adjacent to the inclusion and to test colocalization; however, because these focal planes do not transverse the inclusion lumen, the clear ring-shaped MT cage observed during chlamydial infection (Al-Zeer et al., 2014; Meier et al., 2023; Haines et al., 2021; Dumoux et al., 2015) is not clearly detected here. Moreover, MT structures associated with CPn0572–FLAG signals were often aberrant compared to control MT structures. Specifically, the diameter of MT filaments in the vicinity of the inclusion in control cells was 0.21 µm, whereas MT structures in cells infected with CPn0572-expressing *Ctr* were thicker with 0.278 µm distal of the inclusions and 0.416 µm in proximity to the inclusion (Fig. 5F). When we infected the colon-derived epithelial Caco-2 cells with CPn0572-expressing *Ctr* cells, at 48 hpi CPn0572 filaments and MT filaments were associated and often overlapping, whereas the inclusion lumen was not stained (Fig. 5G), supporting the data obtained for infected HEp-2 cells.

These data show that CPn0572 can be expressed and secreted by *Ctr* and that this strain has a delayed inclusion development. The protein associates with invading EBs and, at 48 hpi, with MT structures, which are thickest near inclusions in comparison to MT structures proximal to inclusions in control cells.

### CPn0572 variants bind MTs directly *in vitro* and stabilize MTs *in vivo*

**Fig. 6.**
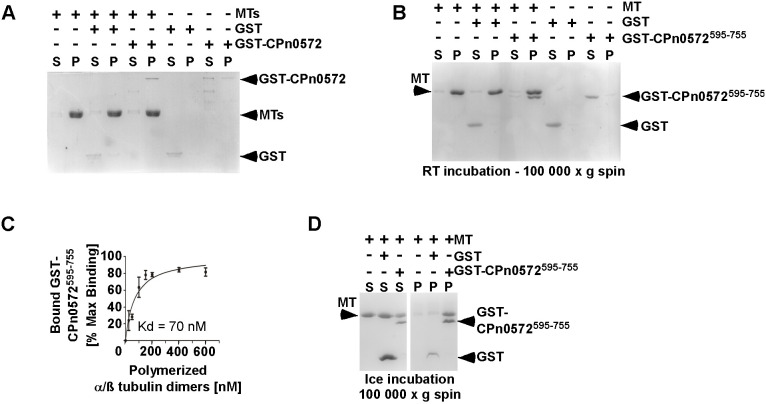
**CPn0572 binds directly to stabilized MTs.** (A) Representative co-sedimentation assay of bacterially expressed GST or GST–CPn0572 incubated with Taxol-stabilized MTs or buffer alone for 30 min at RT. After high-speed centrifugation, matching supernatant (S) and pellet (P) fractions were separated via a 12% SDS-PAGE followed by Coomassie staining. +, present; −, absent. (B) Representative co-sedimentation assay of bacterially expressed GST or GST–CPn0572^595-755^. Experiment was performed as described in A. (C) Equilibrium binding of 100 nM of GST–CPn0572^595-755^ with increasing amounts of Taxol-stabilized MTs in co-sedimentation assays used for the determination of the dissociation constant (*K*_d_) of GST–CPn0572^595-755^ for MTs. Results are mean±s.e.m., *n*=3. (D) SDS gel analysis to determine the resistance of non-stabilized MTs to cold-induced MT depolymerization in the presence of GST or GST–CPn0572^595-755^. All Coomassie-stained gels shown are representative of three repeats.

CPn0572 can associate with and lead to the formation of thicker MTs when expressed ectopically in human cells and during an infection by *Ctr* expressing CPn0572. In addition, the exclusively MT-binding variant CPn0572^595-755^ modulates MTs in a dose-dependent manner. Thus, it was of interest to determine whether CPn0572 could bind MTs directly and to analyze the nature of the MT modulation. To this aim, we purified recombinant bacterially expressed GST and GST–CPn0572 and either incubated it with Taxol-stabilized MTs or with buffer alone. After high-speed centrifugation GST and GST–CPn0572 were found to be predominantly in the supernatant in the absence of MTs (Fig. 6A, four rightmost lanes). In contrast, in the presence of MTs, GST–CPn0572 was exclusively present in the pellet fraction, whereas GST and a faint contaminant band in the GST–CPn0572 preparation remained in the supernatant (Fig. 6A, left and central lanes). We next tested whether purified CPn0572^595-755^ was also able to interact with MTs directly. After high-speed centrifugation CPn0572^595-755^ was found predominantly in the pellet fraction only in the presence of MTs (Fig. 6B). We next tested the strength of the CPn0572^595-755^–MT interaction by determining their apparent equilibrium dissociation constant (*K*_d_). We found that CPn0572^595-755^ bound MTs with an apparent *K*_d_ of 70 nM (95% confidence interval: 26.8 to 113.2 nM) (Fig. 6C). Thus, we conclude that CPn0572 directly binds to MTs.

We next tested the effect of CPn0572^595-755^ on MT stability by incubating GST or GST–CPn0572^595-755^ with Taxol-stabilized MTs on ice. Following a 10 min ice incubation and high-speed centrifugation, depolymerized tubulin subunits were recovered preferentially in the supernatant of MTs alone or MTs incubated with GST (Fig. 6D, three left most lanes). However, MTs incubated with GST–CPn0572^595-755^ were partially protected from ice depolymerization, as evident by their efficient recovery in the pellet fraction (Fig. 6D, three right most lanes).

**Fig. 7.**
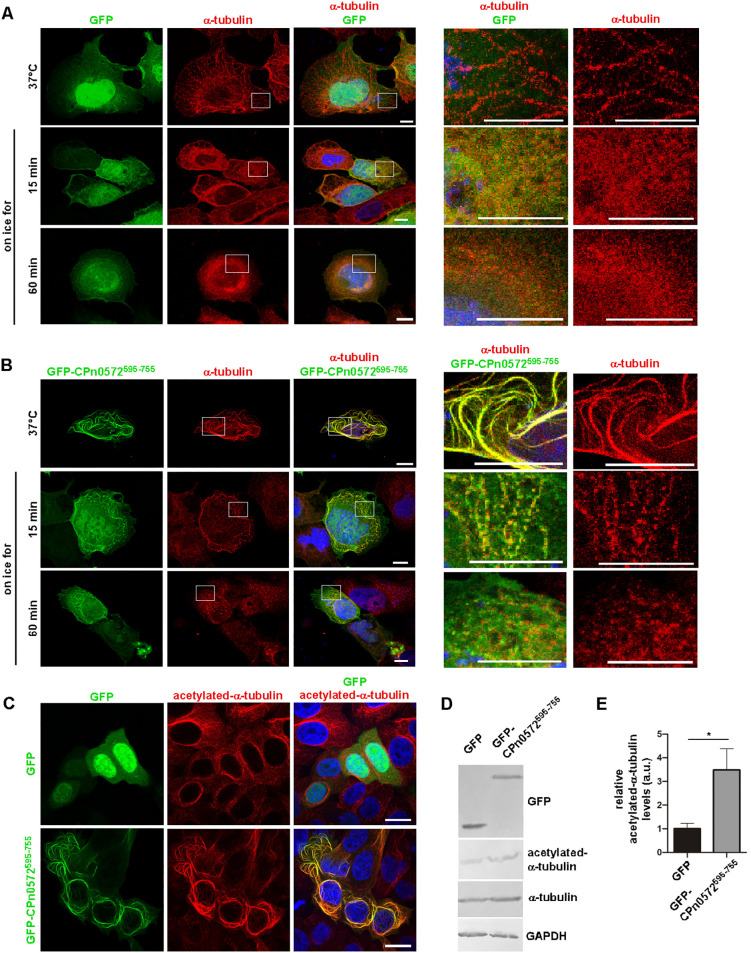
**CPn0572^595-755^ protects MTs against cold-induced MT depolymerization.** (A,B) Representative confocal images of HEp-2 cells transfected with plasmids encoding GFP (A) or GFP–CPn0572^595-755^ (B) for 18 h. MTs were wild-type (after incubation at 37°C) or depolymerized through incubation on ice for 15 or 60 min and then visualized by anti-α-tubulin antibody (red) and DAPI to stain DNA (blue). Boxed regions correspond to the enlarged images shown on the right. Scale bars: 10 µm. (C) Confocal images of HEp-2 cells transfected with a plasmid encoding GFP or GFP–CPn0572^595-755^ for 18 h. MTs were visualized with an anti-acetylated-α-tubulin antibody (red) and DNA with DAPI (blue). Images shown in A–C are representative of three or more repeats. Scale bars: 10 µm. (D) A typical example of a Western blot analysis of HEp-2 cells expressing GFP or GFP-CPn0572^595-755^ for 18 h. Protein extracts were separated on a 10% SDS-PAGE followed by western blot analysis. Western blot was probed with anti-GFP, anti-acetylated-α-tubulin, anti-α-tubulin or anti-GAPDH antibodies. (E) Quantification of relative acetylated-α-tubulin levels shown in D. Band intensities from Western blots of acetylated-α-tubulin in cells expressing GFP–CPn0572^595-755^ were determined and compared relative to control cells (GFP alone=1). ImageJ 1.47v was used for band intensity quantification. Error bars denote ±s.e.m., *n*=3 independent experiments. *P<0.05 (two-tailed unpaired Student's *t*-test).

To determine the impact of CPn0572 on MT characteristics *in vivo*, we expressed either GFP or GFP–CPn0572^595-755^ in HEp-2 cells and incubated them on ice to induce MT depolymerization (Delphin et al., 2012) prior to immunostaining. In control GFP-expressing cells, the MT fibers present prior to ice incubation were barely detectable after 15 min and undetectable after 60 min on ice (Fig. 7A). In contrast, in GFP–CPn0572^595-755^-expressing cells, short MT filament-like structures were still detectable even after 60 min on ice (Fig. 7B). In the latter case the MTs appeared fractured and displayed a punctate pattern that colocalized partially with GFP-CPn0572^595-755^ signals (Fig. 7B, enlargements at the right).

Similarly, in a drug treatment experiment, MTs in GFP–CPn572^595-755^-expressing HEp-2 cells displayed strongly increased resistance to the MT-depolymerization drug nocodazole (Movie 1). Thus, CPn0572^595-755^ has the capacity to stabilize MTs in human cells *in vivo*.

MTs are diversified through a number of posttranslational modifications, which regulate MT properties and are read by cellular effectors, such as molecular motors and MAPs, to provide spatial and temporal specificity to MTs in cells (McKenna et al., 2023). Thus, we wondered whether the stabilization of MTs caused by CPn0572 would be reflected in its acetylation pattern, which has been described as making MTs more resistant to mechanical deformation (Portran et al., 2017). Indeed, in epithelial cells expressing GFP–CPn0572^595-755^ almost all MTs associated with CPn0572 signals, which were thick and abnormally shaped, were acetylated, whereas in GFP-expressing control cells, acetylated MTs are less thick and fewer in numbers (Fig. 7C). Band intensity comparison revealed that in CPn0572-expressing human cells the amount of acetylated α-tubulin was increased 3-fold compared to that seen in control cells (Fig. 7D,E). These data imply that the binding of CPn0572 to MT results in their stabilization and in turn in an increased acetylation.

Although colocalization of full-length GFP–CPn0572 with MTs was only found in a minority of cells, we still wanted to test whether this association was sufficient for MT cold protection. Unlike GFP–CPn0572^595-755^, full-length GFP–CPn0572 was unable to protect MTs after 60 min ice exposure (Fig. S5A). Surprisingly however, we noted a new actin phenotype which was the appearance of actin aggregates that colocalized with GFP–CPn0572 (Fig. S5A–D). We assume that the cells showing actin aggregation are those cells that before ice treatment had CPn0572 on MTs. Considering that depleting cells of MTs has an impact on the actin cytoskeleton of CPn0572-expressing cells, we conclude MTs have the capacity to play an important role in the regulation of CPn0572-mediated actin modulation.

## DISCUSSION

The cytoskeleton of animal cells is a highly complex, adaptable structure required for a wide variety of distinct cellular processes. These range from cargo transport, endocytosis, force transmission and cell division to the processing and subsequent adaptation to extrinsic signals. To carry out these multiple tasks, the actin, MT and intermediate filament networks need to be highly adaptable and communicate with each other. Given the multiple roles of the eukaryotic cytoskeleton, it is unsurprising that the subversion of the infected cells cytoskeleton has evolved in many pathogens and is used for different tasks during the pathogen life cycle (Li et al., 2023; Hartland et al., 2022). For example, Listeria and Shigella use the host actin cytoskeleton for their uptake at the beginning of the infection cycle but also at the end of the cycle to spread from cell to cell (Hartland et al., 2022; Dowd et al., 2021).

The hijacking of the dynamic actin scaffold of the host is also crucial for the pathogenicity of the obligate intracellular bacteria *Ctr* and *Cpn*, both of which represent a worldwide major public health burden (Jordan et al., 2020; Campbell and Hahn, 2020). Here, members of the chlamydial TarP family are known early secreted effector proteins, which nucleate and regulate actin dynamics (Jewett et al., 2006; Caven and Carabeo, 2019). Moreover, TarP signaling activates host signaling proteins such as Rac1, phosphoinositide 3-kinase (PI3K) and the WAVE2 complex, in addition to recruiting the actin effectors formin and Arp2/3 (Romero et al., 2020; Romero and Carabeo, 2022).

In this work, we define an additional and unique role for *Cpn* TarP member CPn0572, beyond its well-established role as an actin nucleator, namely the association with and modulation of the MT cytoskeleton. Several eukaryotic proteins have been identified with the ability to physically crosslink F-actin and MT filaments, among them tau and MAP2 (Cabrales Fontela et al., 2017). However, only formins and adenomatous-polyposis-coli (APC) not only bind MTs and actin, but also have the intrinsic ability to polymerize actin filaments (Juanes et al., 2017; Henty-Ridilla et al., 2016). For instance, the formin mDia1 has the ability to polymerize actin from MT ends. Given that CPn0572 is able to polymerize actin (Jewett et al., 2010) and binds MTs, it is conceivable that this chlamydial effector protein functions in a similar way. Moreover, as these properties are not only found in the human *Cpn* isolate GiD but also in the CPn0572 homolog from the koala isolate LPCoLN, which is phylogenetically basal to the human isolates (Myers et al., 2009), we propose that the dual function of these proteins points to an evolutionarily conserved mechanism.

CPn0572 colocalizes to MTs in human interphase and mitotic cells and binds directly to MTs *in vitro*. Thus, CPn0572 fulfills the characteristics of a MAP. The apparent dissociation constant of CPn0572^595-755^ to MTs of 70 nM is similar to that of tau (Goode et al., 1997; Gigant et al., 2014) and EB1 (Berrueta et al., 1998; Tirnauer et al., 2002).

MTs are highly dynamic structures with the ability to change the MT polymerization–depolymerization cycle according to the needs of the cell. As ectopic expression of CPn0572 in human cells protects MTs from cold- and drug-induced depolymerization *in vivo* and the presence of the C-terminal variant CPn0572^595-755^ leads to increased resistance to cold-induced MT depolymerization *in vitro,* we propose that CPn0572 has a MT stabilization function. How this is achieved on a molecular level still needs to be determined; however, the finding that the presence of CPn0572^595-755^ increases the amount of acetylated MTs in the cell might suggest that changing MT properties could involve tubulin post-translational modification (Janke and Montagnac, 2017).

To our knowledge, CPn0572 is the only bacterial protein identified that directly binds to and regulates both the actin and MT cytoskeletal systems, suggesting that this chlamydial protein might provide a functional crosstalk between the two cytoskeletal systems to modulate specific cellular process(es) (Dogterom and Koenderink, 2019).

What then is the role of CPn0572 in chlamydial infection? CPn0572 is expressed late in the *Cpn* infection cycle so to be present in the infectious *Cpn* EBs, and is detected 15 min post infection on actin and EBs (Jewett et al., 2010; Zrieq et al., 2017; Hänsch et al., 2020). Plasmid-borne expression of CPn0572 in *Ctr* shows that the secreted protein can associate with and modulate MTs in the vicinity of the chlamydial inclusion. The *Ctr* system used is artificial but is a very valuable tool to assay the connection between secreted CPn0572 and MTs. We do not at present know whether the actin-modulating and MT-modulating functions of CPn0572 occur in concert during early infection. However, the two functions appear to be intertwined as shown by the appearance of actin aggregates in cells expressing CPn0572 and a depolymerized MT cytoskeleton.

## MATERIALS AND METHODS

### Construction of plasmids

DNA sequences encoding CPn0572, CPn0572 deletion variants, or other members of the TarP family were amplified by PCR from genomic DNA [*Cpn* GiD, *Cpn* LPCoLN, *Ctr* L2 or *C. psittaci* 6BC; Jantos et al., 1997; Jan Rupp (Department of Infectious Diseases and Microbiology, University of Lübeck, Lübeck, Germany); ATCC no. VR-902B; and Li et al., 2022, respectively] or a pre-existing plasmid (full-length CPn0572, Braun et al., 2019) using custom-synthesized oligonucleotide primers (40-nucleotide homology to expression vector and 20-nucleotide homology to the respective gene) (Merck KGaA, Germany). Amplified DNA sequences were cloned into appropriate plasmids (pEGFP_2xFYVE; #140047 Addgene) with deletion of 2x_FYVE and addition of CEN6-ARSH4-TRP1 cassette from pYD1 (Thermo Fisher Scientific, Waltham, MA, USA), resulting in pAE67; or modified pGEX-5X-1 (GE Healthcare, Chicago, IL) harboring a CEN6-ARSH4-TRP1 where the factor Xa has been replaced with a TEV protease recognition site) using *in vivo* homologous recombination in *Saccharomyces cerevisiae* CEN.PK2 (as described previously; Moelleken and Hegemann, 2008)), amplified in *Escherichia coli* XL-1 blue (Stratagene) and the insertion verified by sequencing (Microsynth Seqlab GmbH, Germany). For CPn0572 expression in *Ctr*, plasmid p2TK2-SW2 IncDProm-RSGFP-IncDTerm (Agaisse and Derré, 2013) harboring an IncD promoter-RSGFP-IncD terminator expression cassette was used as backbone. For cloning by homologous recombination in *S. cerevisiae,* the CEN6-ARSH4-TRP1 cassette from pYD1 (Thermo Scientific, Waltham, MA, USA) was added upstream to the IncD promoter (yielding pKM209) and RSGFP was replaced by a 3× FLAG tag (resulting in pKM255). Finally, the DNA sequence encoding *cpn0572* was cloned N-terminally to the 3× FLAG tag (pMH16).

Final constructs used were: CPn0572: bp 1–2265 (Braun et al., 2019); CPn0572^1-536^: bp 1–1608 (Braun et al., 2019); CPn0572^478-755^: bp 1432–2265 (Braun et al., 2019); CPn0572^536-755^: bp 1606–2265 (Braun et al., 2019); CPn0572^595-755^: bp 1783–2265; CPn0572^615-755^: bp 1843–2265; CPn0572^635-755^: bp 1903–2265; CPn0572^655-755^: bp 1963–2265; CPn0572^806-990^ (*CPn* LPCoLN): bp 2416-2970; TarP^712-870^ (*C. psittaci*): bp 2134–2610; TarP^868-1005^ (*Ctr*): bp 2602–3015.

### Cell culture and plasmid transfection of mammalian cells

Human epithelial HEp-2 (ATCC; CCL-23, contamination free) and U2OS (ATCC; HTB-96, contamination free) cells were cultured in Dulbecco's modified Eagle's medium (DMEM; Thermo Fisher Scientific) supplemented with 10% fetal calf serum (FCS), non-essential amino-acids (Thermo Fisher Scientific) and MEM vitamins (Thermo Fisher Scientific) at 37°C and 6% CO_2_. For transfection, a 70% confluent monolayer was grown in 24-well plates (Sarstedt AG & Co. KG, Nümbrecht, Germany) on coverslips following medium exchange with fresh medium without FCS. After 18 h of transfection using TurboFect (Thermo Fisher Scientific), cells were fixed with 3% paraformaldehyde (Fisher Chemicals) in HBSS for 10 min at room temperature (RT) and washed three times with HBSS (Thermo Fisher Scientific). For permeabilization, 2% saponin (Merck KGaA, Germany) in PBS was used for 20 min at RT. Staining of MTs was performed with anti-α-tubulin antibody (Origene Technologies, Inc., Rockville, MD, USA; #BM753S; 1:150), acetylated MTs with anti-acetylated-α-tubulin antibody (Thermo Fisher Scientific; #32-2700; 1:100), vinculin with anti-vinculin antibody (Merck KGaA; #V9264; 1:100) and actin with Rhodamine–phalloidin as recommended by manufacturer (Thermo Fisher Scientific; #R415; 0.5 µl of 400× stock solution in 200 µl of PBS-Saponin solution for each coverslip), all detected with fluorescently conjugated secondary antibodies (Thermo Fisher Scientific). To visualize DNA DAPI (Merck KGaA; 1:500) was used. For western blot analysis, proteins were verified by using anti-GFP antibody (Thermo Fisher Scientific; #MA5-15256; 1:2500), anti-α-tubulin antibody (Merck KGaA; #T6199; 1:1000), anti-acetylated-tubulin antibody (Merck KGaA; #T7451; 1:20,000), anti-GAPDH antibody (Thermo Fisher Scientific; #MA5-15738; 1:1000).

### Microscopy of mammalian cells and image processing

Images were acquired using an inverse Nikon TiE Live Cell Confocal C2plus with 100× TIRF objective and a C2 SH C2 scanner. All images are displayed as maximum intensity projections, except when noted. Analysis of images and measurements were generated with Nikon Element software and ImageJ 1.47v (National Institute of Health, Bethesda MD, USA).

### Protein purification

GST and GST–CPn0572^595-755^ were expressed in *E. coli* Rosetta (DE3) cells (Merck KGaA) and purified using glutathione agarose beads (Merck KGaA) according to manufacturer's instructions and dialyzed overnight in buffer A1 (50 mM Hepes-HCl pH 7.4, 150 mM NaCl and 20% glycerol; w/v). The purity and integrity of fusion proteins were analyzed by SDS-PAGE and Coomassie Blue staining.

### MT binding and stabilization assays

MT polymerization was undertaken according to the manufacturer's instruction (Cytoskeleton Inc.). MT binding assays were carried out as follows: 25 µM of Taxol-stabilized MTs (equivalent to 4 µM tubulin dimers) in 80 mM PIPES pH 7.0, 2 mM MgCl_2_, 0.5 mM EGTA, 72 µM GTP, 0.4% glycerol (w/v) and 18 µM Taxol were incubated with 25 µl of 4.6 µM GST or GST–CPn0572^595-755^ in Buffer A1 for 30 min at RT. Mixtures were then layered on top of a 100 µl glycerol cushion (80 mM PIPES pH 7.0, 1 mM EGTA, 60% glycerol and 20 µM Taxol) and centrifuged at 100,000 ***g*** at RT for 45 min. The supernatant was carefully collected before the extraction of the glycerol cushion. Pellets were resuspended and loaded next to their corresponding supernatant fraction in a 12% SDS gel, followed by Coomassie Blue staining. For MT stabilization assays, MT polymerization according to manufacturer's instruction was carried out followed by addition of 4.4 µM GST or GST–CPn0572^595-755^ in a final volume of 32 µl. Mixtures were incubated for 25 min at 35°C and then incubated on ice for 10 min. Surviving MTs were stabilized by addition of 28 µl ice-cold taxol-stabilization buffer (80 mM PIPES pH 7.0, 2 mM MgCl_2_, 0.5 mM EGTA and 20 µM Taxol), layered on top of a 100 µl-glycerol cushion, centrifuged at 100,000 ***g*** at RT for 45 min and processed as described above.

### Cold- or drug-induced MT-depolymerization

For cold-induced MT-depolymerization, HEp-2 cells were incubated on ice for 0, 15 or 60 min after transfection. MTs were stained with anti-α-tubulin antibody (Origene Technologies, Inc.; #BM753S; 1:150) and DNA was visualized with DAPI (Merck KGaA, Darmstadt, Germany; 1:500). For nocodazole treatment of HEp-2 cells, a 70% confluent monolayer of HEp-2 cells was grown in a µ-Dish with a glass bottom (ibidi GmbH, Gräfelfing, Germany) for life cell imaging. After 18 h of transfection, cells were washed twice with imaging buffer containing glucose following addition of SiR-tubulin (Spirochrome, Switzerland; #SC002; 1 µM) for MT visualization. Cells were incubated for 30 min at 37°C. DNA was visualized by using Hoechst 33342 (Thermo Fisher Scientific; 1:500). For MT de-polymerization, stained cells were treated with nocodazole (Merck KGaA; 10 µg/ml).

### Infection with *Cpn*

*Cpn* GiD cells (Jantos et al., 1997) was propagated in HEp-2 cells. EBs were purified in a 30% gastrographin solution (Bayer Vital GmbH, Leverkusen, Germany) and stored in SPG buffer (220 mM sucrose, 3.8 mM KH_2_PO_4_, 10.8 mM Na_2_HPO_4_, 4.9 mM L-glutamine) at −80°C. Transfected HEp-2 cells were infected at a given multiplicity of infection (MOI) with *Cpn* GiD by adding purified EBs suspended in DMEM followed by centrifugation at 950 ***g*** at 30°C for 1 h. The medium was replaced by fresh medium containing 12 µg/ml cycloheximide and cells were incubated at 37°C for 30 h. Paraformaldehyde- and methanol-fixed samples were used for microscopy. Inclusions were visualized with the *Cpn* inclusion membrane protein antibody anti-CPn0147 (generated in our lab; 1:50). For determination of internalized EBs, infected cells were fixed with 3% paraformaldehyde after 120 min post infection and uninternalized EBs were stained with a rabbit anti-*Cpn* GiD antibody (generated in our lab; 1:40) prior to cell permeabilization and DNA was stained with DAPI (Merck KGaA; 1:500).

### Plasmid transformation of *Ctr*

A crude stock of *Ctr* L2 was centrifuged for 20 min at 21,885 ***g*** at 4°C. Resuspension of the pellet in CaCl_2_ buffer (10 mM Tris-HCl pH 7.4, 50 mM CaCl_2_) was followed by addition of 5 µg plasmid DNA and incubation for 30 min at RT. The suspension was placed on confluent HEp-2 cells and filled up with fresh medium. After spinning for 60 min at 950 ***g*** at 37°C the medium was replaced fresh medium containing 12 µg/ml cycloheximide (Merck KGaA; #C4859) and incubated over night at 37°C. The next day the medium was replaced by medium with cycloheximide and 1 µl/ml penicillin G (Merck KGaA; #13752) for selection. After incubation for 24 h at 37°C, wells were scraped, sonicated for 30 s and centrifuged for 15 min at 870 ***g*** at 37°C. The supernatant was split up to flasks with confluent HEp-2 cells and centrifuged for 60 min at 950 ***g*** at 37°C. After spinning, the medium was changed with fresh medium with cycloheximide and penicillin G and incubated for 48 h at 37°C. Cells were scraped, sonicated and centrifuged (15 min, 870 ***g***, 37°C). The supernatant was centrifuged for 20 min at 21,885 ***g*** at 4°C. Then the pellet was resuspended in SPG buffer (220 mM sucrose, 3.8 mM KH_2_PO_4_, 10.8 mM Na_2_HPO_4_ and 4.9 mM L-glutamine) in an ultrasonic bath and frozen at −80°C. For protein verification via western blot an anti-CPn0572 antibody (generated in our lab; 1:50), anti-FLAG antibody (Thermo Fisher Scientific, Waltham, MA, USA; PA1-984B; 1:150) or anti-DnaK antibody (from Dr Svend Birkelund, Department of Health Science and Technology, Medical Microbiology and Immunology, Aalborg University, 9220 Aalborg, Denmark; 1:50) was used.

### Infection with transformed *Ctr*

HEp-2 cells were infected by addition of control or plasmid transformed *Ctr* L2 and centrifugation for 60 min at 950 ***g*** at 37°C. The medium was replaced with fresh medium containing 12 µg/ml cycloheximide and cells incubated at 37°C for 15 min. Cells were fixed with methanol, washed two times with HBSS (Thermo Fisher Scientific) and blocked at RT for 10 min with PBS containing 0.1% Triton X-100 and 2% BSA. Cells infected for 48 h were pre-extracted with 80 mM PIPES pH 6.8, 1 mM MgCl_2_, 5 mM EGTA and 0.5% Triton X-100 for 30 s, fixed by addition of 0.5% glutaraldehyde for 10 min and quenched with 0.1% NaBH_4_ for 7 min. Cells were the washed with PBS, and blocked as described above. Anti-FLAG (mouse) antibody (Merck KGaA; #F1804; 1:500) or anti-FLAG (rabbit) antibody (Thermo Fisher Scientific; PA1-984B; 1:500) was incubated overnight at 4°C in blocking solution. Anti-α-tubulin antibody (Origene Technologies, Inc., Rockville, MD, USA; #BM753S; 1:150) was used for MT visualization. DAPI (Merck KGaA, Darmstadt, Germany; 1:500) was used to visualize DNA.

### Phylogenetic analysis

The multiple protein sequence alignment and phylogenetic tree of TarP homologues were generated using the Clustal Omega Multiple Sequence Alignment program (EMBL-EBI) selecting the MBED-LIKE CLUSTERING GUIDE-TREE and MBED-LIKE CLUSTERIN ITERATION.

### Data analysis

For data analysis and statistical significance, Excel (Microsoft Corporation, USA) or Prism (GraphPad Software Inc., USA) was used. A two-tailed unpaired Student's *t*-test was used to determine statistical significance. Sample sizes in experiments were enough to ensure adequate detection of effect size. For figure creation Canvas™ 15 Software (ACD Systems of America, Inc.) was used. No AI tools were used.

## Supplementary Material

10.1242/joces.263450_sup1 Supplementary information
