# Supplementary material for: Direct targeting of host microtubule and actin cytoskeletons by a chlamydial pathogenic effector protein
Source: J Cell Sci. 2024 Sep 6;137(17):jcs263450. doi: 10.1242/jcs.263450 (PMC11444262; doi:10.1242/jcs.263450)
Supplement: Supplementary information [file joces-137-263450-s1.pdf]

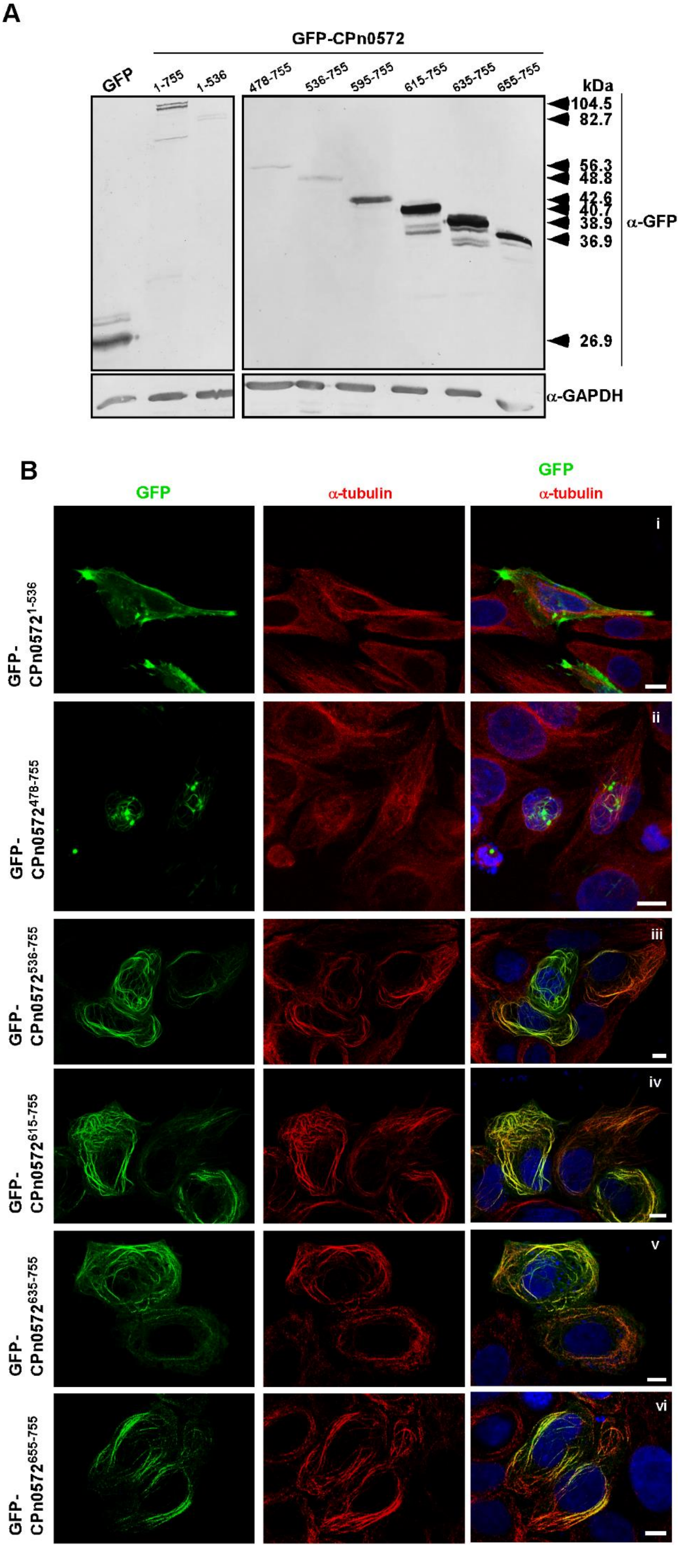

Journal of Cell Science • Supplementary information

Fig. S1. Expression and co-localization of CPn0572 and variants

(A) Western blot analysis of HEp-2 cells expressing GFP or the indicated GFP-CPn0572 protein variants. Numbers indicate amino acids. Proteins were detected on immunoblots probed with anti-GFP or anti-GAPDH antibody. (B) Representative confocal fluorescence images of HEp-2 cells expressing a plasmid encoding the indicated GFP-CPn0572 protein variants. For visualization of MTs an anti-α-tubulin antibody (red) and for DNA DAPI (blue) was used. Scale bars, 10 µm.

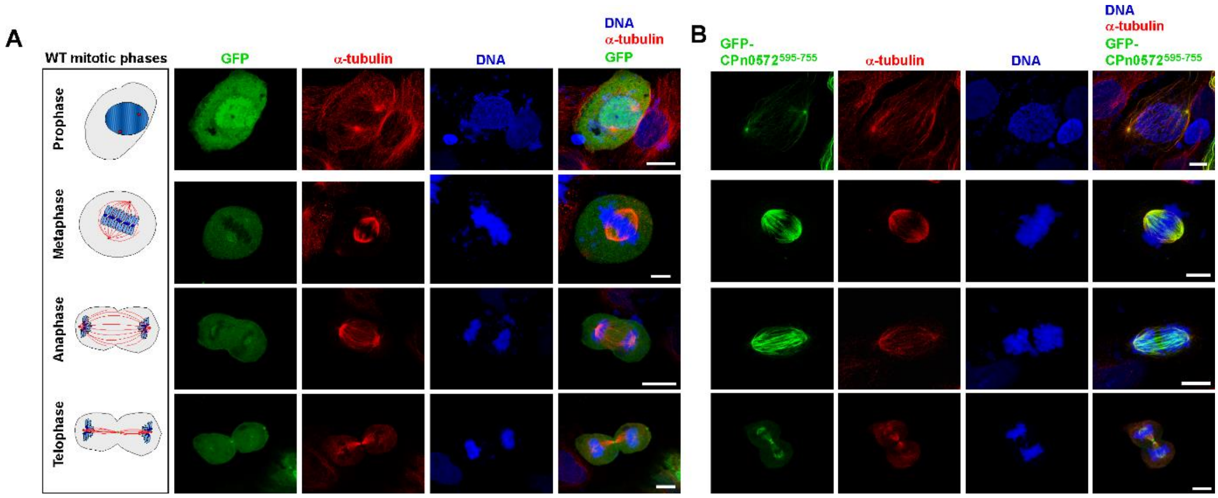

Fig. S2. CPn0572 associates with MTs in mitosis.

(A) and (B) Confocal images of U2OS cells transfected with either a GFP- control plasmid (A) or GFP-CPn0572$^{595\text{-}755}$ plasmid (B) for 18 hrs. Mitotic stages are shown diagrammatically on the left. MTs were visualized by anti-α-tubulin antibody staining (red) and DNA with DAPI (blue). Scale bars, 10 µm.

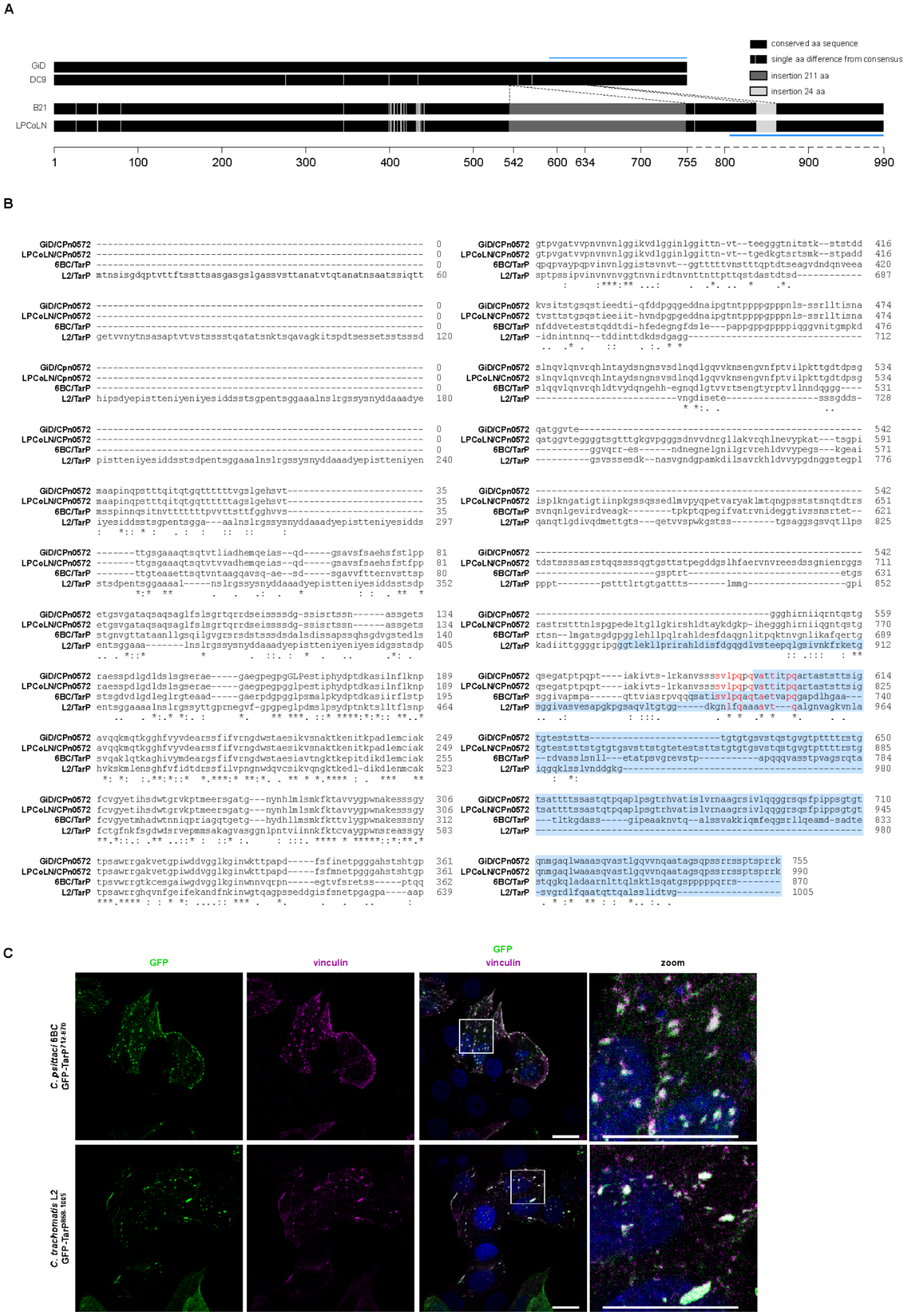

Journal of Cell Science • Supplementary information

Fig. S3. Amino acid sequence and localization analysis of CPn0572 and its homologues from different chlamydial species.

(A) Among 23 CPn0572 homologs from different *Cpn* isolates (Table S1) 20 were identical to the CPn0572 sequence found in the GiD strain. This diagram depicts the amino acid differences of the CPn0572 sequences from the four strains GiD, DC9, B21 and LPCoLN. Black areas indicate identical amino acids, gray lines indicate single amino acid differences, shaded areas denote two blocks of amino acid insertions with dotted lines indicating their position. Blue lines indicate the region utilized for N-terminal GFP-tagged constructs shown in Fig. 2. Numbers at the bottom of the figure indicate amino acid numbers. (B) Amino acid sequence alignment of TarP family proteins from selected *Chlamydia* species (*Cpn* GiD, *Cpn* LPCoLN, *C. psittaci* 6BC and *Ctr* L2) was done utilizing Clustal Omega. Amino acids shown in red depict conserved amino acids in the CPn0572 C-terminus (aa 595 –aa 755) and the C-termini of the other three CPn0572 homologs. The high sequence identity between GiD and LPCoLN CPn0572 sequences allowed a clear identification of a homologous region (highlighted in blue). The amino acid sequences of the C-termini of the CPn0572 homologs from 6BC and L2 have a medium and to low similarity, respectively, and thus larger C-terminal regions were tested to maximize the chances for identification of a potential MT binding domain (highlighted in blue). The selected blue C-terminal sequences of the four species were used in Fig. 2. (C) Representative confocal fluorescence images of HEp-2 cells expressing plasmid encoding *Ctr* L2 GFP-TarP$^{868-1005}$ or *C. psittaci* 6BC GFP-TarP$^{712-870}$. For visualization of vinculin an anti-vinculin antibody (magenta) and for DNA DAPI (blue) was used. Scale bars, 10 µm. White boxes show enlargements.

Journal of Cell Science • Supplementary information

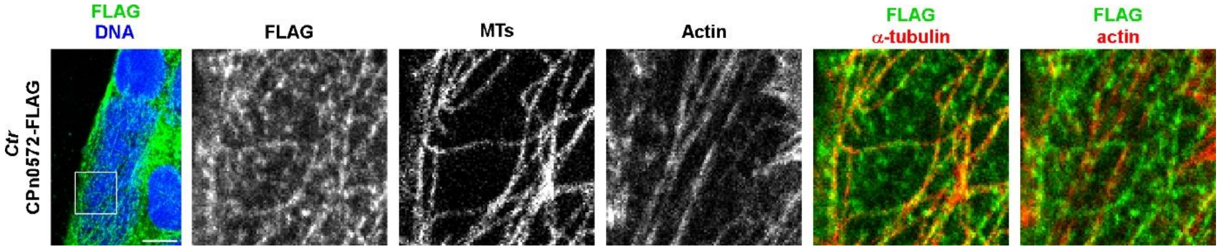

Fig. S4. CPn0572-FLAG expressed in *Ctr* associates exclusively with MTs.

Representative image from 2 independent experiments showing focal planes of HEp-2 cells infected with *Ctr* L2 transformed with a plasmid harboring CPn0572-FLAG (MOI=0.5, 48 hpi). Cells were pre-extracted with Triton for 30 sec and fixed with glutaraldehyde. CPn0572-FLAG was visualized by using an anti-FLAG antibody (green), MTs with anti-α-tubulin antibody, actin with rhodamine phalloidin and DNA of human cells and chlamydial inclusions with DAPI (blue). Images show a focal plane between the inclusion and the plasma membrane. Box shows enlargement. Scale bar, 5 µm.

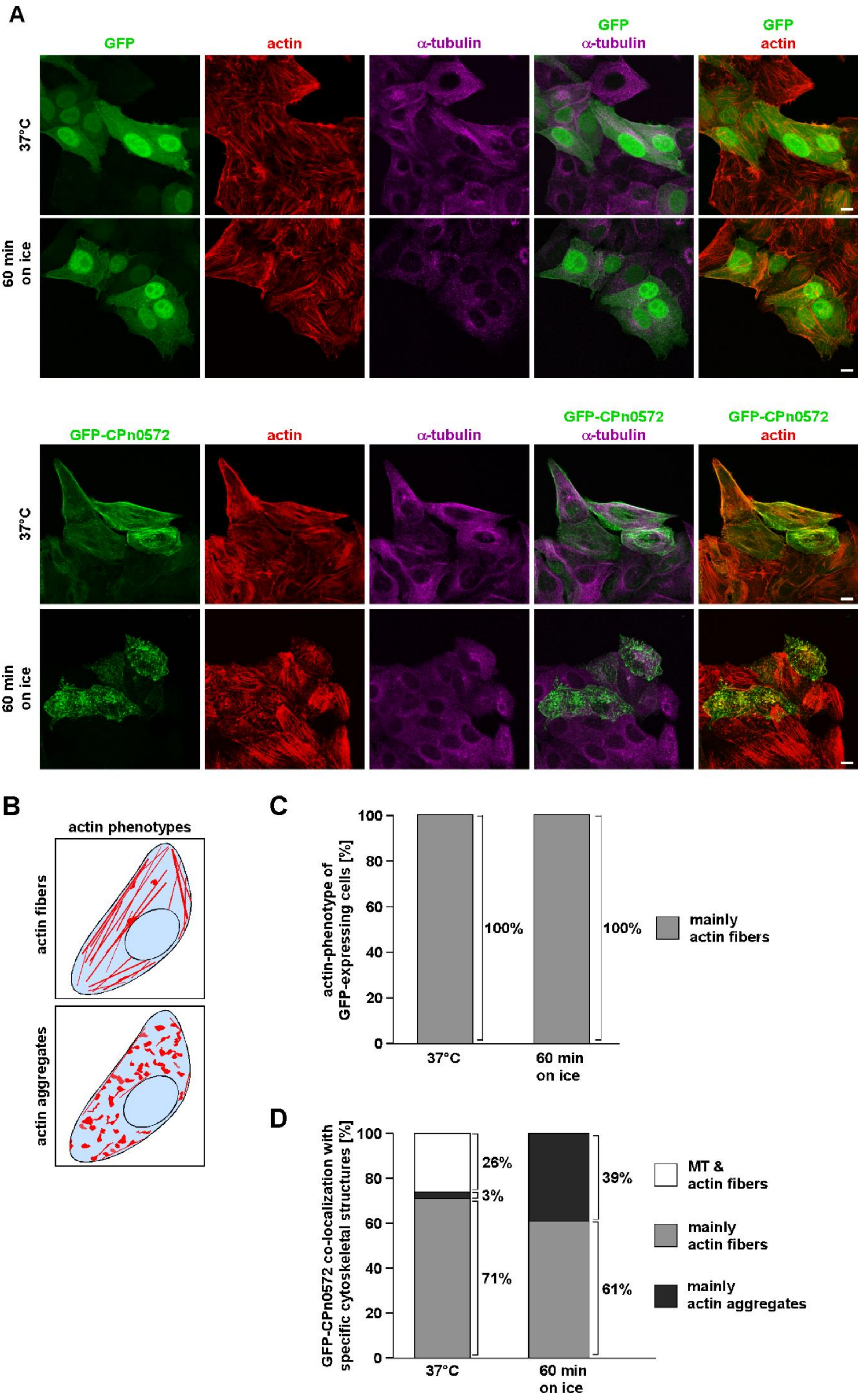

Journal of Cell Science • Supplementary information

Fig. S5. Destabilization of MTs by ice treatment in cells expressing GFP-CPn0572 results in altered actin structures.

(A) Representative confocal images of HEp-2 cells expressing GFP or GFP-CPn0572 (24 hrs) incubated for 60 min at either 37°C or on ice. Actin was visualized with rhodamine-phalloidin (red), MTs with anti α-tubulin antibody (magenta). Scale bars, 10 µm. (B) Schematic representation of the observed actin phenotypes: actin fibers or actin aggregates. (C) Quantification of the actin phenotypes observed in GFP expressing cells kept at 37°C or after incubation on ice for 60 min as described in (A). light grey box, cells showing mainly actin fibers (>60% of the actin structures showing this phenotype). n=1 representing 25 GFP expressing cells. (D) Quantification of the percentage of GFP-CPn0572 expressing cells co-localizing with the indicated cytoskeletal structure at 37°C incubation or after incubation on ice for 60 min. White box; cells showing GFP-CPn0572 co-localization with MTs without/with simultaneous actin fiber co-localization, light grey box; cells showing GFP-CPn0572 co-localization mainly with actin fibers, dark grey box; cells showing GFP-CPn0572 co-localization mainly with actin aggregates. n=2, each representing 50 GFP-CPn0572 expressing cells.

Table S1. CPn0572 gene sequences of 23 *Cpn* isolates.

Chlamydia pneumoniae isolate	GeneBank Reference #	Genomic locus of *cpn0572* [bp]
CWL029	NC_000922.1	662352-664619
AR39	NC_002179.2	176966-179233
J138	NC_002491.1	661673-663940
TW-183	NC_005043.1	659674-661941
Wien1	NZ_LN846980.1	661898-664165
CM1	NZ_LN846995.1	661993-664260
CV14	NZ_LN846996.1	661897-664164
CWL011	NZ_LN847000.1	662350-664617
K7	NZ_LN847221.1	662297-664564
PB2	NZ_LN847241.1	661900-664167
Wien3	NZ_LN847257.1	662347-664614
CV15	LN846999.1	38037-40304
GiD	LN847008.1	661957-664224
MUL2216	LN847227.1	133618-135885
Panola	LN847235.1	46-2313
U1271	LN847244.1	454687-456954
UZG1	LN847245.1	659676-661943
Wien2	LN847254.1	251883-254150
YK41	LN849043.1	14219-16486
H12	LN847189.1	3-2267
B21	AZNB01000021.1	3628-6600
LPCoLN	CP001713.1	176812-179784
DC9	LN847051.1	15607-17874

GeneBank: National Center for Biotechnology Information (NCBI)

Journal of Cell Science • Supplementary information

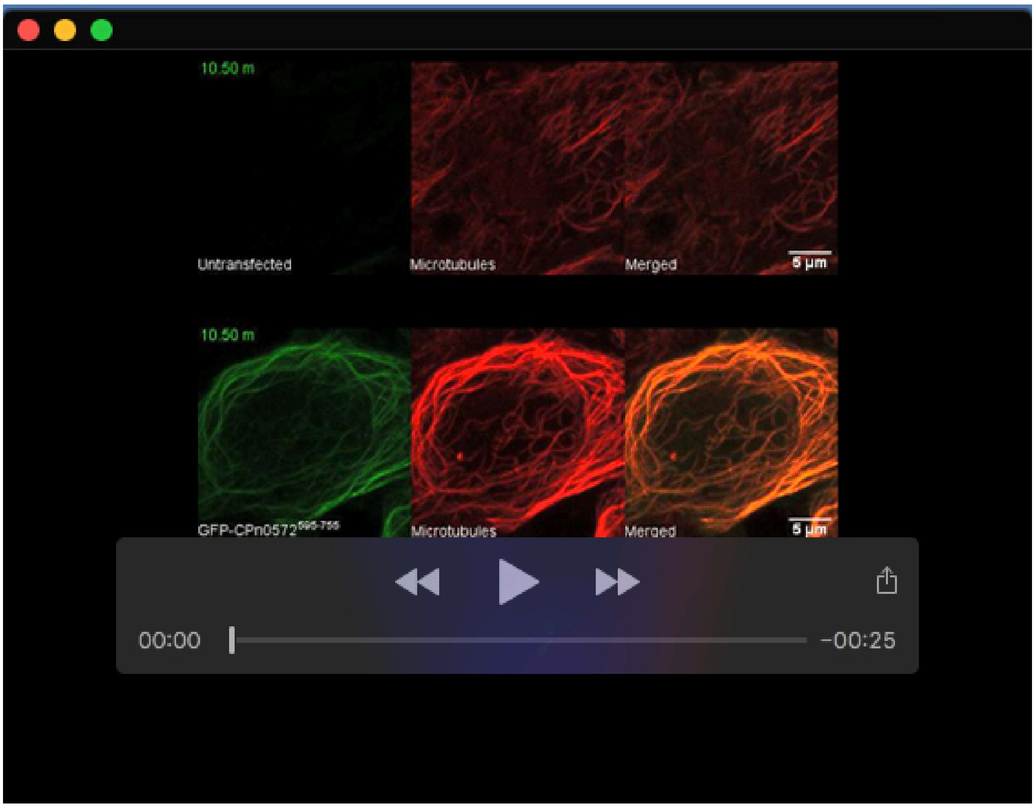

Movie 1. CPn0572$^{595-755}$ expression stabilizes MTs during nocodazole treatment.

Life cell analysis of untransfected HEp-2 cells (top) and HEp-2 cells expressing GFP-CPn0572$^{595-755}$ for 18 hrs (bottom). After staining MTs with SiR-tubulin (red), cells were treated with 10 µg/ml nocodazole to induce MT-depolymerization. Scale bar 5 µm.

Journal of Cell Science • Supplementary information
